# Exploring the Dynamics of Actors, Structural Factors, and Bricolage in the Implementation and Sustainability of eHealth Solutions: Qualitative Multiple-Case Study

**DOI:** 10.2196/79999

**Published:** 2026-01-07

**Authors:** Susanne Eriksen, Christine Øye, Anne Marie Dahler

**Affiliations:** 1 Department of Health and Caring Science, Faculty of Health and Social Science, Western Norway University of Applied Sciences, Bjornsonsgate 45, Haugesund, 5528, Norway, 47 48455062; 2 Faculty of Health and Social Science, Western Norway University of Applied Sciences, Stord, Norway; 3 Department of Applied Welfare Research, UCL University College, Odense, Denmark

**Keywords:** eHealth solutions, innovation processes, interaction, public sector innovation, implementation

## Abstract

**Background:**

European health care systems face mounting pressures from an aging population, workforce shortages, and decentralization, challenging the delivery of accessible, high-quality care. eHealth solutions are widely promoted to enhance efficiency and improve the quality of care. Despite a strong policy report, anticipated benefits remain unrealized, as implementation processes often encounter barriers and high failure rates. Research shows that drivers and barriers are dynamic and shaped by actor interactions. Some studies suggest that certain actors, often acting as bricoleurs, play a critical role in overcoming these barriers through adaptive and improvised practices. However, little is known about how these actors enact roles, what features enable bricolage, and how structural conditions influence these practices.

**Objective:**

The aim of this study is twofold. First, it investigates the roles and features of actors involved in innovation processes, with a particular emphasis on the application of bricolage to overcome barriers and the influence of structural factors on these processes. Second, it aims to contribute both theoretical and empirical insights to deepen the understanding of barrier dynamics within innovation processes.

**Methods:**

We conducted a multiple-case study comprising 10 semistructured interviews, 11 focus groups with health care professionals, managers, trainers, and policymakers, participant observations of training sessions, and document analysis. An iterative process integrated the dramaturgical approach with the concept of bricolage, guiding the reflexive thematic analyses.

**Results:**

Roles were enacted based on available information, context, and assigned functions. Service specialists (eg, superusers) and mediators (eg, unit or project managers) gained backstage insights through shadowing staff, evaluations, and support activities. When mandated and equipped with contextual and technical knowledge, these actors became bricoleurs, addressing unforeseen challenges by creatively mobilizing resources and thereby transforming barriers into promoters. Effective bricolage required proximity to the implementation site, dedicated involvement, and experiential knowledge of health care and technical domains. Key drivers included colocation, supportive management, stable teams, superusers, tailored training, follow-up activities, and informal evaluations. Barriers such as organizational silos, leadership shifts, staffing shortages, high turnover, geographic dispersion, and technology perceived as challenging or surveillance-oriented constrained bricolage and hindered implementation.

**Conclusions:**

Actors may become bricoleurs when their assigned roles, contextual knowledge, and backstage access enable them to improvise in response to unforeseen challenges. Through a dramaturgical lens, bricolage is an adaptive performance that sustains frontstage care delivery. Bricoleurs combine proximity, experiential knowledge, and dual expertise to transform barriers into drivers by adjusting the innovation process and fostering interaction. These practices illustrate the mutual shaping of structure and agency: enabling conditions expand the space for bricolage, while barriers narrow it. Understanding this dynamic is essential for advancing theory on innovation processes and for designing implementation strategies that leverage bricolage as a mechanism for transforming barriers into drivers of innovation.

## Introduction

### Background

One of the significant challenges facing European societies is the aging population, coupled with a shortage of health care staff [1-4]. These challenges are compounded by a deliberate decentralization of health care services, driven by political decisions, which complicates efforts to ensure accessible and high-quality health care [2,5-8]. In response to these pressing issues, there is a growing emphasis on innovation within the public sector [9,10], particularly through the adoption of eHealth solutions [1-3]. eHealth solutions are often defined as the organization and delivery of health services using information and communication technologies (ICTs) to support and enhance health care [4,11]. Across European societies, including Norway and Denmark, which form the context of this study, there is strong optimism that innovative eHealth solutions can improve productivity, free up time for patient-centered care and core health care tasks [2,8,12], and empower older adults to live independently at home by enhancing their health and quality of life [13-16]. The belief stems from the potential that eHealth solutions can enhance efficiency, improve the quality of care, and bolster patient security [17-19]. However, despite a strong policy push for implementing eHealth solutions, authorities acknowledge that the full benefits have yet to be realized [2,3,20,21].

Research on innovation processes involved in implementing and sustaining eHealth solutions in health care reveals that these processes are often fraught with challenges, resulting in a high failure rate [22-26]. Numerous studies have identified a range of factors influencing the outcomes of eHealth innovation processes [22,26-33]. These factors are often presented as separate concepts, being either a driver or a barrier [34,35]. However, scholars are increasingly emphasizing the dynamic nature of drivers and barriers [36-38]. What may initially appear as a barrier can be transformed into a driver if addressed appropriately [38-40]. Detecting barriers and addressing them depends on the interaction among actors as well as proactive engagement from specific actors. Previous research has demonstrated that collaborating actors in innovation processes engage in complex and dynamic interactions and negotiations [38]. As the complexity of the innovation process increases, interactional barriers become more prominent [34,36,41], hindering effective knowledge sharing, which is crucial for implementing and sustaining eHealth solutions [25,32,38,42,43]. Research has identified specific actors who are effective in overcoming interactional barriers [38]. Such actors are frequently found to act as boundary spanners, bridging knowledge gaps, for example, between ICT staff, management, and health care professionals. By bridging knowledge gaps, these actors can transform barriers into opportunities, making innovations more contextually relevant and beneficial by, for example, facilitating the redesign of workflows, providing adequate training and support to users, and highlighting problematic issues [26,31,38,44]. These actors can also be referred to as bricoleurs [45-47], and their contributions are considered vital for integrating eHealth solutions into health care practices [7,48,49]. Bricolage is characterized by its spontaneous and improvised nature, involving small, pragmatic adjustments in response to unforeseen events or emerging needs [47,49]. This process is often informal, builds on embodied experience within the practice, and is inherently collaborative, as it necessitates interaction with other actors [45,47,49]. Given its intangible nature, it remains unclear how the context can facilitate bricolage work [46]. There also remains an incomplete understanding of the nature of the roles of these bricoleurs as well as how these roles might be identified, enabled, and enhanced [26,36,38,49,50]. The literature emphasizes the importance of investigating individual actors as units of analysis, given that microlevel practices have been largely overlooked [24,30,50]. Research has identified various features of individual actors involved in implementing and sustaining innovations, including ICT-related skills, experience, demographics, and personality traits [26,35]. Creative and empowered actors, who possess the ability to navigate and overcome a risk-averse administrative culture, play a crucial role in driving innovation [35]. However, it remains unclear how these features interact with the enactment of roles and the contextual factors. Bricolage provides an approach that emphasizes how innovation can be made possible by recognizing both the work of actors and the influence of structural factors [47]. We therefore consider the concept of bricolage useful for understanding the complex dynamics between the micro- and system levels of innovation processes.

### Objectives

Against this background, this study aims (1) to investigate the roles and features of actors involved in innovation processes, with particular emphasis on the application of bricolage to overcome barriers and the influence of structural factors on these processes; and (2) to contribute both theoretical and empirical insights to deepen the understanding of barrier dynamics within innovation processes. We pursue these aims through a qualitative, multiple-case study set within a European Union innovation project that focuses on enhancing digital skills and innovation readiness. By examining 3 cases of implementing and sustaining innovative eHealth solutions, our study seeks to address the following research questions:

- How do actors involved in implementing and sustaining eHealth solutions enact the role of bricoleurs, and what features enable this role?
- In what ways is bricolage performed to transform barriers into drivers of innovation, and how do structural conditions shape or constrain these practices?

## Methods

### Study Design

The study used an exploratory case study design that adhered to the principles of multiple holistic case study design [51]. Each case served as a distinct unit of analysis. The exploratory case study is an empirical investigation that examines processes and uncovers mechanisms related to a contemporary phenomenon within its real-life context [51,52]. This approach was well-suited to our research design, as we aimed to understand the roles of various actors in innovation processes and to investigate how they overcome barriers and transform them into drivers of innovation, as well as how structures influence the enactment of roles and performance of bricolage.

### Case Selection and Data Collection

The cases selected for this study were part of the Digital and Innovation Skills Helix project, a European Union initiative aimed at enhancing digital skills and promoting innovation readiness. As part of a forthcoming innovation process aimed at implementing an eHealth solution, these cases tested 3 tools developed through the Digital and Innovation Skills Helix project. These tools facilitated the acquisition of digital skills, cocreative implementation planning, evaluation, and competence assessment. Further details about the tools can be found in our previous work [53].

The 3 cases are situated in a Nordic welfare context. Case A is a nursing home situated in a rural municipality in Western Norway, offering 24-hour health and care services to residents for both short-term and long-term stays. This municipality actively participates in regional and national eHealth networks and collaborates closely with neighboring municipalities to enhance health care services. The nursing home accommodates 41 residents and employs 60 staff members, including 1 leader and 4 unit managers. Since 2017, the municipality has systematically upgraded its outdated equipment with new eHealth solutions, such as electronic medicine dispensers and safety alarms. At the time of data collection, the nursing home implemented a new patient monitoring system that included digital supervision. This system’s primary objectives were to increase service efficiency and improve patient security.

Case B is a home care service located in a rural municipality in Western Norway, providing 24-hour assistance with daily living and home health services. Most service users are frail older adults who require support to continue living at home. This home care service supports approximately 150 service users and employs 50 health care staff, including the home care service unit manager. Since 2015, the municipality has focused on implementing eHealth solutions in the health care sector to address demographic challenges and deliver sustainable health care services. The home care service implemented electronic door locks (e-locks) in service users’ homes, aiming to provide faster and safer assistance.

Case C is a cross-sectoral collaboration between a hospital and a municipal home care service in Southern Denmark. The hospital offers emergency care, outpatient treatment, and examination services to patients who have been injured. The home care service offers 24-hour assistance, including support for daily living and somatic and psychiatric care. In 2018, the local hospital initiated a project to collaborate with the municipality through video consultations, specifically for discharging complex and vulnerable patients from the hospital to municipal care. Aligned with the Regional Council’s digitalization strategy, the objective was to enhance cross-sectoral collaboration using technology, streamline discharge conferences, and ensure more coherent patient care. The hospital was allocated approximately US $1.5 million to develop digital competencies among its staff.

The selection of cases was pragmatic, as the available cases within the European Union project were limited, resulting in the inclusion of 3 cases that exhibited varying levels of complexity. As case C involved implementing video consultations in a cross-sector collaboration between a hospital and a municipal home care service in Southern Denmark, this case is more complex compared to cases A and B, which were more similar and less complex, as they implemented an eHealth solution within a single organization, engaging only a limited number of employee groups. The empirical material for these cases included document analysis, participant observation, semistructured individual interviews, and focus groups (see Multimedia Appendix 1 for an overview of the data collection).

The interviews and focus groups, with some being more prominently featured than others, served as a central component of our analysis, providing in-depth insights into the experiences and perspectives associated with the implementation process (Multimedia Appendix 2). The participant observations took place during training, where the observer participated and observed questions, discussions, and task-solving activities. The participant observations complemented the interviews, enhancing the analytical depth of the study by offering a comprehensive understanding of the setting and context as well as nuanced insights into the social dynamics (Multimedia Appendix 3). Although these observations, along with the document analysis, served as critical supplementary materials, they primarily provided a contextual backdrop for interpreting the qualitative data derived from interviews and focus groups. The data collection took place between 2020 and 2022, during and after the testing phase of the 3 tools. The data were gathered in person or online via Zoom (Zoom Video Communications) or Microsoft Teams, conducted by SE, AMD, or CØ. The data collection ended when data saturation was reached.

Participants for the study were recruited with the assistance of key stakeholders involved in implementation planning and training within the clinical settings. In cases A and B, a designated project manager from each case served as our primary point of contact. For case C, we contacted the individuals responsible for training at the hospital, where one of the trainers became our primary point of contact. These 3 contact persons facilitated the recruitment process by reaching out to potential participants by email. A total of 949 individuals were identified as potential participants from the training roster, and 31 individuals were identified from the implementation working groups. We contacted 115 individuals, of whom 39 agreed to participate in the interviews, and 70 agreed to participate in participant observations, resulting in a final sample of 109.

### Ethical Considerations

Ethics approval was obtained from Sikt—Norwegian Agency for Shared Services in Education and Research (ref: 198584). Before participation, all participants were provided with informed consent that they signed before participation, which included a comprehensive overview of the study project, its objectives, and data handling procedures. No compensation was provided to the participants. Anonymity was ensured by replacing names with pseudonyms and revising the empirical material to obscure recognizable quotes. To ensure data security, the original recordings as well as transcripts were stored as password-protected files on a secure research server, with access limited to the three authors only.

### Analyses

NVivo (version 21; Lumivero) software was used to analyze verbatim transcriptions of audio-recorded interviews and focus groups. The reflexive thematic analyses for this study were applied to generate initial themes by identifying patterns of shared meaning across the dataset [54-56]. We followed the 6-phase approach developed by Braun et al [55]. First, we aimed to enhance reliability in the analysis process by conducting independent readings of the transcripts. Second, all three authors generated initial themes independently, guided by the concept of bricolage [57] in combination with the dramaturgical approach [58] and the Consolidated Framework for Implementation Research [59]. Since the study aimed to investigate role enactment, bricolage strategies, and structural factors, we approached the data, exploring and tagging text that reflected strategies involving bricolage activities and role features akin to those of mediators and service specialists, as well as structural factors affecting implementation. Third, all three authors constructed themes through thematic mapping, which involved visually exploring potential themes and subthemes, as well as connections between them. Finally, themes were revised and defined in collaboration between all three authors before producing the report. Researcher reflexivity was actively pursued in accordance with the standards of qualitative research [60]. This included engaging in critical dialogue to challenge and complement each other’s interpretations and explicitly acknowledging our personal and professional backgrounds early in the research process. These discussions helped us remain aware of our initial assumptions and avoid conflating prior perspectives with insights emerging from the data.

### Conceptual Framework

The dramaturgical approach by Goffman [58] illustrates how actors manage their frontstage performances within social settings to navigate and influence others’ perceptions. In contrast, backstage involves actions that support the frontstage performance but do not align with its presented image. This perspective highlights that inventive strategies and adaptive behaviors emerge in response to the interactions and perceptions within actors’ immediate social contexts rather than being dictated solely by structural conditions. By integrating the dramaturgical approach and the concept of bricolage, we aim to develop a more comprehensive understanding of how actors can transform barriers into drivers through performing bricolage. Previous research has indicated that distinct roles, such as mediators and service specialists, are pivotal in overcoming interactional barriers [38]. In the paragraph below, we delve deeper into the roles of mediators and service specialists, emphasizing their essential function in bridging interactional gaps.

A mediator acts as an intermediary, facilitating mutually beneficial agreements between 2 potentially opposing teams. By cultivating trust and managing confidential information, the mediator maintains a delicate balance, sometimes projecting a skewed perception of loyalty to foster closeness and understanding among the teams. Examples of mediators may include facilitators, project leaders, or department managers. Conversely, service specialists focus on constructing, repairing, and maintaining performance. Acting as “scene workers,” they enable actors to effectively perform their roles and define situations without encountering dramaturgical obstacles [58]. Examples of service specialists could include ICT specialists, champions, or “superusers.”

To investigate the strategies used by mediators and service specialists in merging frontstage with backstage to overcome barriers and further the innovation process, we integrate Lévi-Strauss’s [57] concept of bricolage. Bricolage, derived from the French verb “bricoleur,” means to tinker or improvise. Lévi-Strauss [57] conceptualized bricolage as a creative method of using available resources, contrasting the improvisational nature of the bricoleur with the systematic, planned approach of the engineer. This duality draws attention to different problem-solving methods [57]. Since its introduction, bricolage has found relevance across various disciplines [61-63], including studies on public sector innovation [7,45-49,64]. While extensive research has examined bricolage activities, there has been less focus on bricoleurs themselves [49]. This oversight may stem from Levi-Strauss’s [57] structuralist perspective, which suggests that objective structures shape actors’ lives, often overshadowing individual interpretations. However, latter interpretations of the concept of bricolage are not strongly underpinned by the structuralist approach and see bricolage as an activity where the bricoleur creates structures from resources at hand [46]. As such, bricolage is considered relevant for capturing the dynamics of innovation and the connection between microlevel practices and broader systems of innovation [46]. Along with other researchers [65-67], we argue for the need to develop new approaches to advance public sector innovation processes. As such, to better understand the motivations and strategies of actors, it is necessary to recognize the interplay between different approaches. Situated in the critical realism paradigm, we acknowledge that both Goffman’s and Lévi-Strauss’s approaches can complement one another. While objective structures influence individual agency, especially in complex situations, actors’ agency is also shaped by their subjective interpretations of social reality.

The dramaturgical approach illuminates the social dynamics and interpersonal interactions on the microlevel that shape how bricoleurs operate, for example, how the roles are enacted, negotiated, and adapted based on the context and audience. Conversely, bricolage enhances our understanding of the resourcefulness and adaptability that actors demonstrate in their roles, particularly in overcoming barriers to innovation. It elucidates how bricoleurs creatively respond to unforeseen events posed by the context while simultaneously negotiating and navigating structures that influence their actions.

## Results

### Overview

The total sample consisted of 109 participants, including 39 who participated in interviews and 70 who participated in participant observations. The participants represented a diverse range of stakeholders, including health care professionals, unit managers, leaders, policymakers, ICT specialists, eHealth solution providers, trainers, project staff, and technical personnel. The following sections outline the themes, accompanied by illustrative quotations.

### Enacting the Role of a Bricoleur

The actors assigned the role to spearhead the innovation processes in the 3 cases were a project manager (case A), a home care service manager (case B), and a project manager assisted by trainers (case C). According to Goffman [58], roles are enacted based on available information, the situational context, and the function an actor is expected to perform. When assigned a function such as project manager (case A and C), home care service manager (case B), or trainer (case C), actors face specific expectations from their audience, which they strive to meet or engage in impression management to convey that these expectations are being met. In case B, the role of a home care service manager entailed numerous time-consuming operational responsibilities, which constrained the time and attention available for the implementation process—particularly during the COVID-19 pandemic. The geographical distance also prevented him from shadowing staff and observing their activities firsthand. These conditions made it challenging to enact either a mediator or a service specialist role. Compounding these issues, the home care service manager was new to the organization, starting the position only 2 weeks after the national lockdown in Norway. These circumstances complicated the enactment of a bricoleur role. First, the manager lacked familiarity with the organizational context and staff. Second, not having participated in the planning phase, he had limited background knowledge of the e-lock project’s rationale and objectives, as reflected by the following quote: “It was decided before I started working here. So, I’m not entirely sure of the background [of the project], but it’s probably to save time on key usage and to increase accessibility and safety for the users” (Respondent 1, home care service manager). Finally, the operational demands inherent in the managerial role overshadowed the implementation process, leaving little opportunity to prioritize it. This contrasts with cases A and C, where project managers were fully dedicated to the implementation process and exempt from other operational responsibilities. Although the project managers were fully dedicated to the innovation process, the way it unfolded varied across the 2 cases. In case A, the project manager and a project coordinator moved into the nursing home during the initial 2 weeks of implementation, providing continuous on-site support and guidance. After this period, they remained available via telephone and email and maintained an office next door to ensure ongoing support and rapid problem-solving. Due to poor collaboration with the ICT department in the municipality, the project manager and coordinator were also compelled to take on responsibilities as ICT specialists. This additional responsibility led the project manager and the coordinator to enact roles of service specialists, which had many advantages for the ongoing process, as expressed by a staff member:

*I think it would have been difficult without them. It makes the workday easier. We spend less time on frustration, because, well, technology isn’t my field, you know. My field is actually healthcare. But having those who are a support function for technology somehow makes my day easier*.

[Respondent 2, nurse]

As the patient warning system and digital supervision were implemented directly within the nursing home, the service specialists remained in proximity to the site of action. The continuous presence of the project manager and coordinators, who engaged in shadowing staff and observing daily routines, enabled them to adopt dual roles as both service specialists and mediators gradually. In doing so, they effectively bridged the gap between backstage and the front stage. Drawing on their familiarity with the context and the actors involved, along with their acquired ICT competencies, they gradually enacted roles as bricoleurs—constructing, repairing, and maintaining the health care professionals’ performance so they could do their “actual job” (Respondent 2, nurse). The project team also trained a group of superusers, who in turn took on roles as service specialists and actively supported bricolage activities by drawing on their digital competence.

Although the project manager in case C was committed to the innovation process, the physical location—outside the hospital in a separate building—created a spatial distance from the implementation site. Furthermore, the affiliation with a research and innovation unit, rather than the hospital units or the municipality, meant that the project manager lacked an established foothold within the everyday workplace dynamic. These conditions challenged the ability to enact a role as a mediator or service specialist, as the project manager was not embedded in the daily routines of the health care staff and thus had limited access to backstage information, such as training needs and perceptions about the video consultations. Enacting roles as mediators and bricoleurs was also challenging for the unit managers when priorities competed, tasks were incompatible, and responsibilities conflicted. As expressed by a unit manager: “We were a COVID unit, so we already had plenty to just ... work through, so the idea of creating new ideas and having the time to implement new systems ... Well, the staff was also overloaded. You reach a certain limit where you can’t take in any more” (Respondent 3, hospital unit manager). However, in some of the units, the trainers were able to step into roles as service specialists and bricoleurs when they were physically present in the hospital to support the setup of video consultations. Their role as trainers granted them access to the backstage where health care professionals prepared for their frontstage performances. This backstage access enabled the trainers to observe real-world challenges and tailor both the training and video consultations to the specific needs of each unit. Drawing on their ICT expertise, they engaged in bricolage, creatively assembling and adjusting tools and practices to support the health care professionals in delivering care. The combination of service specialist and bricoleur roles illustrates how these actors not only facilitated the technical implementation but also helped maintain the integrity of the health care professionals’ frontstage performance. As reflected by the hospital director:

*Every time we introduce a new ICT or digitalisation product, we turn the experts into novices. They deal with new technology, altering patient-health professional relationships* [...]. *If the doctor is struggling with an ICT system, they lose some respect in the eyes of the patients. Because then the patient sees them fumbling around and may think they are equally clumsy with all their professional expertise*. [...] *How can we ensure our healthcare professionals are not novices in ICT but at least able to use it effectively, so they don’t come across as incompetent or anything like that? When healthcare professionals experience technical problems, they tend to revert to what they are accustomed to and can handle better.*

[Respondent 4, hospital director]

This quote highlights the delicate balance between adopting eHealth solutions and maintaining a professional identity. Trainers who acted as bricoleurs helped preserve this balance by ensuring that health care professionals could maintain confidence and competence in their frontstage roles, even when navigating unfamiliar eHealth solutions.

The 3 cases show that the ability to enact the role of bricoleurs depends on actors’ experiential knowledge, proximity to the implementation site, and a dedicated focus on implementation, which makes backstage dynamics more accessible.

### Performing Bricolage to Transform Barriers Into Drivers

Goffman [58] distinguishes between 2 models of behavior: the real and the contrived. The real is regarded as a genuine performance, which is not consciously assembled, but an unintended product of an actor’s spontaneous reaction to the actual situation at hand. Contrived performances, on the other hand, are regarded as carefully constructed, with each artificial element added one by one, since the behavior is not reacting to any actual situation. How one responds spontaneously to unforeseen events determines whether the performance is characterized by bricolage or a more systematic and planned engineering approach to overcome barriers [57]. Refer to Table 1 for an overview of the drivers and barriers across the 3 cases.

**Table 1.** Cross-case synthesis of structural drivers and barriers of bricolage.

Structural factor	Drivers	Barriers
Organizational structure	Colocation and small teams enable rapid feedback and access (A); proximity in small municipalities fosters collaboration (A, B).	Silos and interorganizational differences hinder coordination (A, C); concurrent large IT projects (electronic health records) compete for attention (C); geographic dispersion (A, B, C); role overload in small municipalities (A, B).
Leadership and governance	Supportive management and dedicated project leadership drive progress (A, B, C).	Shifting leadership and lack of clear ownership delay implementation (B, C); operational pressures crowd out strategic work (C).
Political framework	Alignment with local or regional or national priorities; digitalization targets create mandate (A, B, C).	—^a^
Resources	Adequate funding and equipment (A, B, C); stable workforce (A, B); superusers support adoption (A).	Staffing shortages, turnover, and crises (eg, COVID-19, strikes) impede adoption (C); lack of designated superusers (B, C).
Cultural norms and values	Positive work culture (A), and perceived expectations to use technology motivate uptake (A, B, C).	Strong patient-safety ethos and professional identity can slow change (A, B, C); small-community dynamics can amplify resistance (A).
Training and competence development	Needs-based, one-on-one, learning-by-doing, and follow-up (A, B, C) are effective; dedicated trainers also provide support (A, C).	24/7 operations complicate scheduling (A, B, C); generic courses without local tailoring are less effective; trainer distance and high turnover reduce retention (C).
Infrastructure and technology	Technology perceived as applicable or simple supports use (A, B); technology used proximate to superusers or project management supports use (A).	The technology is implemented in settings that lacked proximity to superusers and management (B, C). Technology perceived as surveillance (A), time-consuming (B, C), poorly functioning (C), and not beneficial (B, C) hinders its use. Poor set-up (A, C) and lack of deimplementation (B) create friction.
Communication systems	Multiple channels (meetings, emails, direct access to project leads) aid communication (A, B, C).	Weak interdepartmental and sectoral channels (A, C) and 24/7 staffing patterns (A, B, C) limit the information flow; the existing communication system between sectors hinders the development of a new communication system (C).
Daily relations and collaboration	Colocation and bridging roles (eg, unit management and superusers) improve collaboration (A).	Geographic dispersion (A, B, C) and lack of cross-sector workflows impede collaboration (A, C).
Quality assurance and evaluation routines	Routine monitoring (B, C) and frequent informal evaluation support technology adoption (A).	Monitoring can be perceived as surveillance; the absence of informal feedback loops reduces responsiveness (B, C).

a Not applicable.

By being proximate and shadowing the staff, the project manager in case A was present when unforeseen events happened. For example, when a ghost appeared on the patient monitoring system, as elicited by the project manager:

*We set up* [digital supervision] *for a user* [and] *it was supposed to alert us if the person got out of bed*. [...] *It’s nighttime, and the user is restless, so a night shift comes in* [...]. *She sits on the edge of the bed to calm the patient down. Then she stands up, which triggers an alert for getting out of bed because she’s probably been sitting on the bed for too long. Due to our poor Wi-Fi, the signal is delayed*. [...] *Then, a few minutes pass, and an alert goes off on the mobile phones that the bed is abandoned. An anonymized picture of a man appears. Then, the others panic and rush in, wondering who’s here, and they get the idea that this is a ghost* [...]. *They find out that the woman’s late husband is in the room. Because he lived at the nursing home before.*

[Respondent 5, project manager]

One of the health care professionals:

[...] *There’s probably much more between heaven and earth than we see, but I’m unsure if it would affect patient monitoring*. [...] *It’s sometimes a profession shrouded in superstition and stories*. [... ] *We are often close to death, right? So maybe it’s a bit natural to become a bit superstitious.*

[Respondent 2, nurse]

Even though the project manager could not convince the staff that the ghost appeared due to a slow Wi-Fi signal, responding constructively to this event spontaneously required an understanding of the context and the actors involved, as well as technical competence to grasp how cultural and emotional factors intersect with technological implementation. The project manager also participated in staff meetings to evaluate the use of the patient monitoring system, ensuring that issues were raised and resolved. The training was conducted informally and continuously, based on immediate needs:

*We simply have to practice. I have to show the staff and test. I don’t know how often I’ve been on the floor to demonstrate a fall. [...] I have to show them and have them try it themselves. We constantly have to repeat, demonstrate, you know* ... “*Now, I triggered a violence alarm. What do you do then?” And it’s like* ... *recreating scenarios.*

[Respondent 5, project manager]

By being present and practicing in the situation, the project manager made it challenging for the staff to conceal actions or project desired impressions. This led the staff to use the patient warning system and digital supervision even when they were not fully confident in operating it. They either invited the project manager backstage for assistance or proceeded without the necessary skills, which created technical challenges and unforeseen events. These events, however, functioned as dramaturgical disruptions that exposed latent vulnerabilities in the performances of health care professionals. Once these issues were made visible, the project manager could implement improvisational strategies using the available resources.

In contrast to case B, where the e-locks were used in service users’ homes, this made it difficult for the home care service manager to maintain proximity and shadow health care professionals during their work. Consequently, the home care service manager was not exposed to many unforeseen events caused by technology. The e-lock system log was used to monitor use, but it did not provide sufficient information to facilitate bricolage. As a result, the home care service manager remained unaware of how the staff perceived the e-locks. According to the staff, they only used the e-locks about 50% of the time. One staff member had experienced the e-lock malfunctioning once, so they always carried the regular keys “just in case” (Respondent 6, nurse). Staff members did not convey their experiences and concerns. Consequently, the management remained unaware of these issues. As reflected in the quote from the home care service manager below:

It was straightforward and a truly positive thing. It probably hasn’t caused many obstacles. Of course, some actors might have hesitated a bit more to carry it out, but it’s not like they have rallied others, because it’s so simple. So I haven’t heard that anyone has really resisted or that there has been noise around this technology. I would have known if there had been something.

[Respondent 1, home care service manager]

This quote reflects that when new or inexperienced leaders are given formal authority over more experienced staff members, the formally empowered actor often enacts a role of symbolic dominance. In contrast, the staff members are the ones who truly run the show [58]. This discrepancy between formal authority and practical influence served as a barrier to performing bricolage. Transforming this barrier to a driver requires strategies to gain backstage access. Because 24/7 operations made formal training difficult, he improvised by installing an e-lock on his office door, allowing health care professionals to practice at their convenience. This arrangement enabled him to remain close to the action and shadow staff during their practice sessions—providing a form of backstage access. These efforts ensured that staff acquired the necessary competencies to operate the e-lock. The staff found the training sufficient for using the e-locks, as remarked: “It wasn’t that difficult” (Respondent 6, nurse). However, the staff questioned the usefulness and the functionality of the e-locks, as reflected in the following exchange:

 Respondent 6, nurse: *If* [the e-lock] *works, it’s certainly easier.*

 Respondent 7, nurse: *Hmm ... I’m not so sure about that.*

 Respondent 8, nurse: *If it works.*

 Respondent 7, nurse: *It’s very easy just to find the right key and unlock it.*

 Respondent 8, nurse: *Yeah, especially if it’s raining.*

As such, this performance preparation training facilitated by the home care service manager had a limited effect, as e-locks were still not consistently used in service users’ homes. The home care service unit manager emphasized the importance of managing explicit resistance happening on the frontstage: “Those who I thought might give the most resistance, they were actually the first ones I gave training to, and maybe followed up a little extra” (Respondent 1, home care services manager). However, silent backstage resistance might be a barrier more challenging to overcome than explicit frontstage resistance. In case C, many units struggled to implement video consultations for discharge conferences; however, a few units succeeded by engaging in adaptive, improvisational practices. In these units, the technology was not merely adopted as prescribed but reinterpreted and repurposed for alternative use—such as municipal rehabilitation supervision or admission meetings with relatives—illustrating a shift from the scripted “frontstage” plan to context-sensitive enactments. Training similarly moved backstage, taking place within the units rather than in formal classroom settings, fostering unforeseen events and situated learning. The trainers emphasized the importance of readily available support to answer questions and assist with new technology, as stated in the following quote from one of the trainers, highlighting the need for continuous and organized follow-up training in the units:

[...] *Sometimes I think we could try to be more organised in training in the units. I’m not sure if there should be someone who becomes a superuser or is somehow responsible for it* [...]. *If one were to mention a missing link, I think it’s the transition from having received the training to becoming an integrated part of the unit. That’s where we could do something more*. [...] *It works really well if they have had training with us, and then we have been* [in the units]

[Respondent 9, trainer]

The performance of bricolage in these units was facilitated by the trainers’ presence backstage, their flexibility, and consistent availability for support, as well as their provision of ad hoc problem-solving for unforeseen events. As the 3 cases show, responding to unforeseen events in a systematic and planned manner can be challenging. To effectively perform bricolage, it is essential to understand and act upon the context and engage with the involved actors. Understanding the context derives from experiential knowledge, the function assigned, and access to backstage insights. Performing bricolage is not just about creatively navigating unforeseen events using the resources at hand; it is also about being mindful of the interactions and relationships with other actors, ultimately shaping the experience and outcome for everyone involved in the innovation process.

### Features of a Bricoleur

Several common features were observed among the actors who performed bricolage. They were familiar with the local work context, able to move fluidly between technical and clinical domains, and demonstrated a readiness to improvise and adapt eHealth solutions to meet the practical needs of health care professionals. As the project leader in case A noted: “Now I am the ICT department. I go into the computer cabinet myself and connect things” (Respondent 5, project manager). Another feature of the bricoleurs was their assigned role, such as project leader or trainer, which required full dedication to the innovation process. This meant they could be consistently available, responsive, and actively worked to meet the expectations of both the leaders and the health care professionals they supported, as emphasized by one of the trainers in case C: “It doesn’t matter how much time has passed; I always make myself available” (Respondent 9, trainer). The physical proximity to the implementation site and involved actors enabled frequent, informal interaction that granted them access to backstage areas.

In contrast, actors who lacked the essential features for performing bricolage, such as those who were physically distant from the implementation site or held a position that involved other tasks and responsibilities, struggled to enact roles as bricoleurs. Without embeddedness in the local work context or access to backstage interactions, for example, by being new to the organization, these actors were less able to understand the nuances of everyday practices or respond to emerging needs. For instance, this resulted in staff perceptions of management being “unresponsive to feedback” (Respondent 7, nurse) in case B or feeling surveilled as in case C:

[The management] *is definitely monitoring whether we are conducting these video consultations. We figured that out. We didn*’*t actually know. But they are keeping an eye on us*. [...] *What is the reason for that? Well, I think it’s because we first tried to say* ... “*Do we really need this? And should we do it now?” It was very emphatically stated that we should. Also, somewhere between the lines, it says that most consultations should be conducted over video. We have internally determined that as long as we say half/half, we’ll have to see if they eventually knock on the door and say “no, no, no, now there are too many physical meetings every day”*

[Respondent 10, nurse]

This quote captures the management’s attempt to gain backstage access without success, while the nurse and her team strive to protect this backstage area. Consequently, actors without bricoleur features struggle to gain backstage information and are left to resort to more systematically planned approaches, limiting their contributions to, for example, formal training sessions or top-down strategies with limited impact on everyday practices among health care professionals.

Key features of bricoleurs are their familiarity with the local context, the ability to bridge technical and clinical domains, and consistent presence at the implementation site. Together with their dedicated roles and informal access to backstage dynamics, they are enabled to improvise and adapt eHealth solutions to meet practical needs.

### Interactions Between Structures and Actors

In all 3 cases, the implementation of eHealth solutions was aligned with local, regional, and national priorities. The initiatives were supported by committed leadership, adequate funding, and a shared perception among actors that the use of eHealth solutions was both expected and necessary. However, these drivers carried limited weight when other structural factors (see Table 1 for an overview of barriers and drivers across cases), such as shortages of health care professionals, posed substantial barriers to implementation, as expressed by a member of the executive hospital management in case C, which was also the case featuring the most complexity:

*We want to do a lot, but we don’t have the resources*. [...] *We hear: “ You get a lot of money; just get started.” On the other hand, we simply don*’*t have anyone who can* [do it]. *We clearly see that there is a need for these things*. [...] *It creates a vicious cycle spiralling downward, and we need to turn it around somehow.*

[Respondent 4, hospital director]

Other barriers to bricolage included organizational silos (case C), interorganizational (case A) differences, shifting leadership (cases B and C), the pressures of 24/7 operations (all cases), high turnover rates (case C), the absence of superusers (cases B and C), and a strong patient-safety ethos (all cases). Additional barriers stemmed from the maintenance of professional identities (all cases), a lack of deimplementation practices (case B), the absence of informal feedback loops, and geographic dispersion among the actors. Technology itself also posed barriers, particularly when it was perceived as a form of surveillance (case A), as time-consuming (cases B and C), or as offering limited benefits to clinical practice (cases B and C). Under such conditions, performing bricolage becomes a challenging task.

Despite structural complexity and barriers, bricolage occurred across all 3 cases. Actors were not merely shaped by these structures; they actively worked within and upon them. Actors performed bricolage, particularly when structural drivers created openings for creative and adaptive problem-solving. While organizational silos, 24/7 operations, and technological infrastructure influenced what was possible, actors leveraged their assigned functions and interactions, using their expertise to adapt, reshape, and, at times, transform these structures. Key drivers of bricolage included colocation and proximity between actors, which facilitated informal interactions and backstage access. The presence of supportive management, dedicated project leads, and a stable workforce created a foundation for continuity and responsiveness. A high number of superusers, along with tailored training that addressed the specific needs of each unit, enabled health care professionals to engage more confidently with eHealth solutions. Follow-up activities, dual roles (where project leaders also serve as ICT support), routine monitoring, and frequent informal evaluations further supported backstage access and, consequently, adaptive and creative problem-solving.

## Discussion

### Principal Findings

This study explored how actors involved in implementing and sustaining eHealth solutions enact roles as bricoleurs and perform bricolage to transform barriers into drivers of innovation. Using Goffman’s [58] dramaturgical approach and Lévi-Strauss’s [57] concept of bricolage, we examined how structural factors and actors’ agency interact in complex innovation processes.

This cross-case analysis showed that the ability to enact the role of a bricoleur increased when actors were assigned a function dedicated to the innovation process, thereby being freed from operational demands. For example, being assigned the function of a trainer (case C) provided dedication, but also a mandate and a set of expectations to be fulfilled. Further, the enactment of the bricoleur role depended on actors’ experiential knowledge and proximity to the implementation site, which facilitated access to backstage dynamics. The project team in case A is a good example that demonstrates how proximity and presence facilitated backstage access where routines were rehearsed, problems surfaced, and informal knowledge was shared. This backstage access led to a deeper understanding of the everyday challenges faced by health care professionals. Due to the project teams’ continuous presence, health care professionals had to use eHealth solutions despite technical uncertainty, which in turn led to genuine performances, flaws, and unforeseen events. These findings resonate with previous research on bricolage [45,47,49] and add to the incomplete understanding of how the bricoleur role can be identified, enabled, and enhanced [26,36,38,49,50].

Bricolage emerged as a spontaneous and improvised response to unforeseen events, as found in previous research [47,49]; however, our research expands the literature by showing that bricolage builds on the bricoleur’s experiential knowledge and dual expertise in clinical and technical domains, as was apparent in cases A and C. In these cases, the bricoleurs could transform barriers such as 24/7 operational demands and technological issues into drivers through, for example, ad-hoc, tailored training and by adapting solutions in contextually appropriate ways. Previous research has highlighted that barriers are dynamic and may be transformed into drivers if addressed appropriately [36-40]. We propose that bricolage offers a promising approach for facilitating such transformations.

Without backstage access, actors are left to rely on formal and systematic plans, strategies, and communication, as was the case for the project manager in case C and the home care service manager in case B. In both cases, silent resistance in the form of subtle, unspoken disengagement with the eHealth solutions hindered the implementation. Unlike explicit resistance, silent resistance may be difficult to detect and address, especially when actors lack backstage access. While previous research has emphasized how interactional barriers can hinder innovation processes and underscored the importance of collaboration [25,32,34,36,38,41-43] and boundary-spanning roles [26,31,38,44] in mitigating such barriers, our study adds nuance by demonstrating how bricoleurs can mitigate interactional barriers through backstage access.

Despite structural and contextual constraints, bricolage emerged across all cases. In case A, due to collaboration challenges with the ICT department, the project manager acquired ICT competencies and assumed responsibility for ICT-related tasks, which ultimately proved beneficial to both the staff and the innovation process. Rather than being passively shaped by structural conditions, actors can interact with and actively influence the conditions. This highlights how bricolage is not only resourceful but also interactive and performative, enabled by the interplay between agency and context, offering insight into the previously noted uncertainty regarding how the context facilitates bricolage work [46].

Our findings align with and enrich existing implementation frameworks such as the Non-adoption, Abandonment, Scale-up, Spread, and Sustainability framework [25,68] and Consolidated Framework for Implementation Research [59]. While these frameworks emphasize the importance of context, complexity, and actor engagement, they lack a detailed account of the microlevel improvisations that sustain implementation in practice. We contribute to an underexplored area [24,30,50] by proposing that bricolage offers a complementary mechanism that explains how actors navigate complexity not by eliminating it, but by working within and around it. As such, we suggest that bricolage can be conceptualized as a mechanism that links structural factors with microlevel practices (eg, improvisation, adaptation, and role enactment). This perspective can inform the design of implementation strategies that are more responsive to local contingencies and the agency of actors.

Our study aligns with previous literature on the characteristics of actors driving innovation, highlighting the importance of experience and ICT-related skills [26,35]. However, our study expands the literature by demonstrating that features of a bricoleur also encompass proximity, dedication to the implementation process, dual-expertise, and access to backstage dynamics. These features were most evident in cases A and C. As such, the findings of our study demonstrate that bricolage is not a spontaneous phenomenon or a matter of individual creativity [35]; rather, it is contingent upon various structural factors and interactions with actors. Our contribution offers nuanced, empirically grounded, context-sensitive illustrations that enrich existing understandings of bricolage and its role in innovation processes [45-49]. Although prior studies have conceptualized bricolage [45-49], our research advances the field by identifying the conditions and actors that enable its practical enactment. Our research advances the field by empirically demonstrating how specific structural and contextual conditions, such as proximity and dedicated roles, enable the positioning of actors to enact roles as bricoleurs. Through detailed cross-case analysis guided by dramaturgy and bricolage, we have identified key features of bricoleurs and demonstrated how backstage access enables them to respond to unforeseen events in real time. This responsiveness creates opportunities to transform barriers into drivers, thereby sustaining innovation processes in complex health care settings.

In times of austerity, bricolage—an approach that uses available resources—may be notably applicable. Specifically, given that public sector innovation processes are often fraught with challenges and have a high failure rate [22-26], and as new approaches to advance, public sector innovation processes are asked for [65-67].

### Limitations and Future Research

This study used a qualitative multiple-case design with methodological triangulation. While this approach enabled a rich and nuanced understanding of the implementation of eHealth solutions, the findings are grounded in 3 specific cases in a Nordic welfare context. As such, transferability to other settings may be limited. However, the implementation of eHealth solutions in health care is highly relevant across a wide range of countries. eHealth solutions tend to influence professional roles, interactions, and organizational structures in ways that are difficult to predict and anticipate [32,38,42,43,69]. These dynamics apply regardless of context. While our findings are not intended to be generalized, the conditions that enable successful bricolage—such as proximity and dual expertise—may offer valuable insights across diverse contexts.

Future research could build on this work by using mixed methods designs or larger-scale comparative studies to examine how bricolage unfolds across different health care systems or policy environments. Additionally, further exploration of silent resistance, including its manifestations, consequences, and strategies for mitigation, could deepen our understanding of the subtle dynamics that shape innovation processes in complex health care settings.

### Conclusions

This study contributes to the understanding of innovation processes in the public sector by illuminating how actors navigate complex implementation processes through bricolage. By integrating dramaturgy with bricolage, we offer a novel analytical lens that captures both the performative and improvisational dimensions of innovation work. This theoretical pairing enabled us to explore how roles are dynamically enacted and adapted in response to structural and contextual factors, as well as emergent challenges.

Our findings show that actors become bricoleurs not merely by individual traits, but through a combination of contextual knowledge, proximity to the implementation site, and access to backstage dynamics. These conditions enable bricoleurs to improvise, adapt, and sustain innovation efforts in ways that formal strategies alone may not achieve. Our study provides detailed, context-sensitive illustrations that enrich existing theories of innovation and implementation. Recognizing and supporting conditions for bricolage may help design more adaptive, responsive, and sustainable strategies. We demonstrate how bricolage operates as a mechanism that links structural factors with microlevel improvisation, offering a valuable complement to established implementation frameworks. The theoretical pairing further clarifies how social expectations, role performances, and backstage interactions shape the conditions under which bricolage can occur.

## Supplementary material

10.2196/79999 Multimedia Appendix 1 Overview of data collection.

10.2196/79999 Multimedia Appendix 2 Interview and focus group guide.

10.2196/79999 Multimedia Appendix 3 Participant observation guide.

## Abbreviations

e-locks: electronic door locks
ICT: information and communication technology
