# Supplementary material for: Exploring the Dynamics of Actors, Structural Factors, and Bricolage in the Implementation and Sustainability of eHealth Solutions: Qualitative Multiple-Case Study
Source: J Med Internet Res. 2026 Jan 7;28:e79999. doi: 10.2196/79999 (PMC12779101; doi:10.2196/79999)
Supplement: Multimedia Appendix 1 [file jmir-v28-e79999-s001.docx]

**Table S1. Overview of data collection.**

This table summarizes the data collection process for each case, detailing the number and roles of participants, the methods employed for data gathering, and information on the duration and execution of interviews and observations. Additionally, it provides information on who conducted each data collection method.

| **Data collection** | **N =** | **Role of participants** | **Type of method** | | **Duration** | **Researcher** | **Conducted** |
| --- | --- | --- | --- | --- | --- | --- | --- |
| **Case A** | **A nursing home in Norway implementing a patient warning system and digital supervision** | | | | | | |
| Observation | 12 | Nursing home manager  Unit managers (3)  Healthcare professionals (4)  Project manager  Project coordinator  eHealth solution providers (2) | Participant observation | 1 h | | SE | April 2022 |
| Interview 1 | 1 | Project manager | Individual interview | 40 min | | SE | September 2021 |
| Interview 2 | 6 | Nursing home manager  Unit managers (3)  Healthcare professionals (2) | Focus group | 38 min | | SE | April 2022 |
| Interview 3 | 1 | Project coordinator | Individual interview | 45 min | | SE | April 2022 |
| Interview 4 | 1 | Healthcare professional | Individual interview | 31 min | | SE | May 2022 |
| Interview 5 | 1 | Healthcare professional | Individual interview | 50 min | | SE | November 2022 |
| **Case B** | **A home care service in Norway implementing electronic door locks** | | | | | | |
| Interview 1 | 2 | Project coordinator  Department manager of health | Focus group | 1 h 26 min | | SE | October 2021 |
| Interview 2 | 1 | Homecare service manager | Individual interview | 30 min | | SE | July 2022 |
| Interview 3 | 3 | Healthcare professionals (3) | Focus group | 58 min | | SE | August 2022 |
| Interview 4 | 3 | Department manager of technical operations  Janitor | Focus group | 49 min | | SE | October 2022 |
| **Case C** | **A hospital and home care service in Denmark implementing video consultations** | | | | | | |
| Observation 1 | 15 | Municipal nurses (6)  Hospital nurses (6)  Trainers (2)  Hospital IT specialist | Participant observation | | 7 h | AMD | August 2020 |
| Observation 2 | 11 | Hospital nurses (8)  Trainers (2)  Hospital IT specialist | Participant observation | | 7 h | AMD | November 2020 |
| Observation 3 | 15 | Municipal nurses (6)  Hospital nurses (6)  Trainers (2)  Hospital IT specialist | Participant observation | | 7 h | AMD | November 2020 |
| Observation 4 | 11 | Hospital nurses (8)  Trainers (2)  Hospital IT specialist | Participant observation | | 7 h | AMD | March 2021 |
| Observation 5 | 6 | Hospital nurses (3)  Trainers (2)  Hospital IT specialist | Participant observation | | 4 h | AMD | May 2021 |
| Interview 2 | 1 | Municipal nurse | Individual interview | | 30 min | AMD | October 2020 |
| Interview 3 | 1 | Nurse | Individual interview | | 30 min | AMD | October 2020 |
| Interview 4 | 1 | Nurse | Individual interview | | 30 min | AMD | December 2021 |
| Interview 5 | 3 | Trainers (3) | Focus group | | 53 min | SE, AMD | November 2021 |
| Interview 6 | 3 | Unit manager  Nurses (2) | Focus group | | 1 h 21 min | SE, AMD | November 2021 |
| Interview 7 | 1 | Nurse | Individual interview | | 47 min | SE, CØ | November 2021 |
| Interview 8 | 1 | Unit manager | Individual interview | | 40 min | SE, CØ | November 2021 |
| Interview 9 | 4 | Municipal leaders (3)  Municipal IT specialist | Focus group | | 57 min | SE, CØ | November 2021 |
| Interview 10 | 4 | Municipal middle managers (2)  Municipal case worker  Telehealth consultant | Focus group | | 41 min | SE, CØ | November 2021 |
| Interview 11 | 3 | Hospital director  Policymakers (2) | Focus group | | 53 min | SE, AMD | November 2021 |
| Interview 12 | 2 | Unit manager  Nurse | Focus group | | 46 min | SE, AMD | November 2021 |
| Interview 13 | 2 | Nurses (2) | Focus group | | 55 min | SE, AMD | November 2021 |
