# Supplementary material for: Exploring the Dynamics of Actors, Structural Factors, and Bricolage in the Implementation and Sustainability of eHealth Solutions: Qualitative Multiple-Case Study
Source: J Med Internet Res. 2026 Jan 7;28:e79999. doi: 10.2196/79999 (PMC12779101; doi:10.2196/79999)
Supplement: Multimedia Appendix 2 [file jmir-v28-e79999-s002.docx]

**The interview starts with questions related to participant characteristics (around the table if focus group):** Can you describe the tasks and responsibilities you have in your workplace? How long have you had your current position? What is your professional background? What is your age?

**Questions:**

1. Can you tell me about the eHealth solutions you have been trained in or use during your workday?

2. What has been the greatest success when it comes to the implementation and use of telehealth technology in the unit? Can you tell me about a project or collaboration that went well? What happened? Why did it work so well? Can you tell me about a project that did not go so well?

3. Do you use eHealth solutions during your workday? (Why not?) In what way do eHealth solutions affect your workday?

4. What is the biggest value of using eHealth solutions?

5. What is the biggest challenge of using video eHealth solutions?

6. What do you do if the eHealth solutions do not work as they should?

7. Who do you collaborate with when you use eHealth solutions? How do you collaborate?

8. Have you been involved in the planning of the implementation of the eHealth solutions, or the planning of the training? How have you been involved?

9. Have you participated in any of the planning meetings? How did you experience that? What worked? What did not work? Do you have any suggestions for changes?

10. How did you experience the eHealth solutions training? How did it take place? What worked? What did not work so well? Do you have any suggestions for changes?

11. In what way has the training influenced the way you use eHealth solutions?

12. Have you gone through any form of evaluation after the training? How did the evaluation take place?

13. How is the atmosphere in the organization or the hospital unit? Is there anything particular influencing the use of eHealth solutions positively or negatively?

14. How does the management create an environment that is enabling or constraining the use of eHealth solutions?

15. What kind of support do you get within the organization or outside the organization that makes a difference in the implementation and the use of eHealth solutions?

16. We would like to end the interview by asking; What does it take to succeed with the implementation of eHealth solutions? What is the key to success? Or the opposite? What can others learn from your experiences?

17. Is there anything else you would like to add?
