# Supplementary material for: Exploring the Dynamics of Actors, Structural Factors, and Bricolage in the Implementation and Sustainability of eHealth Solutions: Qualitative Multiple-Case Study
Source: J Med Internet Res. 2026 Jan 7;28:e79999. doi: 10.2196/79999 (PMC12779101; doi:10.2196/79999)
Supplement: Multimedia Appendix 3 [file jmir-v28-e79999-s003.docx]

| **Location** |  |
| --- | --- |
| **Time and date** |  |
| **Observer** |  |
| **Number of participants present** |  |
| **Number of organizational staff/trainers present** |  |
| **Telehealth technology** |  |
| **Duration** |  |

**Introduction**

The purpose of the observation is explained before the observation begins. As such, the observer will follow the participants during the on-the-job training course, because we want to gain insight into how the on-the-job training works in practice. Further, to detect themes through the observations that may be interesting to elaborate in interviews. After the observation, there may be a small summary where the participants can clarify whether there is anything they want to explain or add, etc. The topics below are tentative and broad and related to the tools in the education intervention.

**Relevant areas of focus:**

Trainers (project leaders, organizational staff)

- How do the trainers greet the participants? (What is said and done? What terms are used? Etc.)
- How is the plan for the training course presented? (Is the plan in written form? Where? What does it say?)
- How are the learning outcomes presented?
- Is the value of the technology to the end-user, health care professionals, and the organization presented? How is it presented?
- Is the training material in written form? Where is it, and what does it say?
- How do the trainers negotiate with the healthcare professionals participating in the course? How do the trainers respond to comments from healthcare professionals? Hvordan forhandler læringsfasilitator med helsepersonell som skal ha opplæring?
- What learning methods are used? (cases, hands-on-training, dialogue-based, reflection)
- Are the participants actively involved in the training?
- Does the training contain problem-solving (where the technology does not work as it should), and how does the problem-solving take place?
- How do the trainers assess that the participants have acquired the knowledge, skills, and competencies they need to use the technology in practice?
- Is there any evaluation? How does the evaluation take place?

Participants (healthcare professionals):

- What do the participants do when they enter the location? What do they say, and where do they place themselves?
- Who do the participants address? (other participants? Trainers?)
- How do the participants behave toward each other?
- How do participants respond when trainers address the participants? (verbal and body language)
- How do the participants talk about telehealth technology? (values, barriers, opportunities?)
- What do the participants seem to be occupied with during the training? The specific telehealth technology?
- How do the participants engage in the training?
- How do they respond to the training activities? (Verbal and body language)
- Are the participants resistant to training activities? How?

**Facilities:**

- Location
- Interior
- Training facilities
- Equipment
- Training material (manual, guide, checklist)
- Refreshments
